# Supplementary material for: Diallel panel reveals a significant impact of low-frequency genetic variants on gene expression variation in yeast
Source: Mol Syst Biol. 2024 Feb 14;20(4):362–73. doi: 10.1038/s44320-024-00021-0 (PMC10987670; doi:10.1038/s44320-024-00021-0)
Supplement: Supplementary file 1 — Appendix [file 44320_2024_21_MOESM1_ESM.pdf]

Appendix

for

Diallel panel reveals a significant impact of low-frequency genetic variants on gene expression variation in yeast

Andreas Tsouris[1], Gauthier Brach[1], Anne Friedrich[1], Jing Hou[1], Joseph Schacherer[1,2]

1. Université de Strasbourg, CNRS, GMGM UMR 7156, Strasbourg, France
2. Institut Universitaire de France (IUF), Paris, France

This document includes:

Appendix figures S1-S4 and legends (page 2-5)

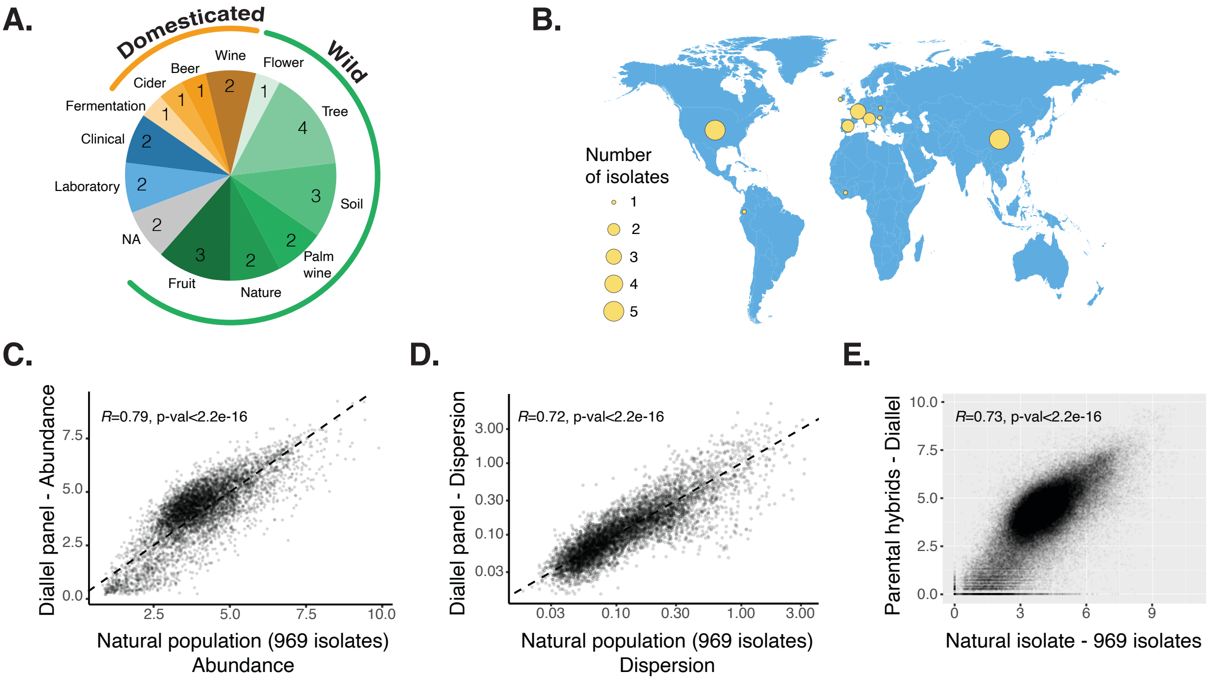

Appendix figure S1. Selection of the parental isolates and correlation between the transcript abundance in the diallel and the natural population

(A, B) Ecological and geographical origin of the parental isolates that were used to generate the diallel crossing panel. Pearson's correlation of the average transcript abundance (C) and dispersion (D) for each gene between the diallel population and the population of 969 natural isolates. (E). Pearson's correlation of the transcript abundance of each homozygous hybrid and the respective homozygous diploid natural isolate.

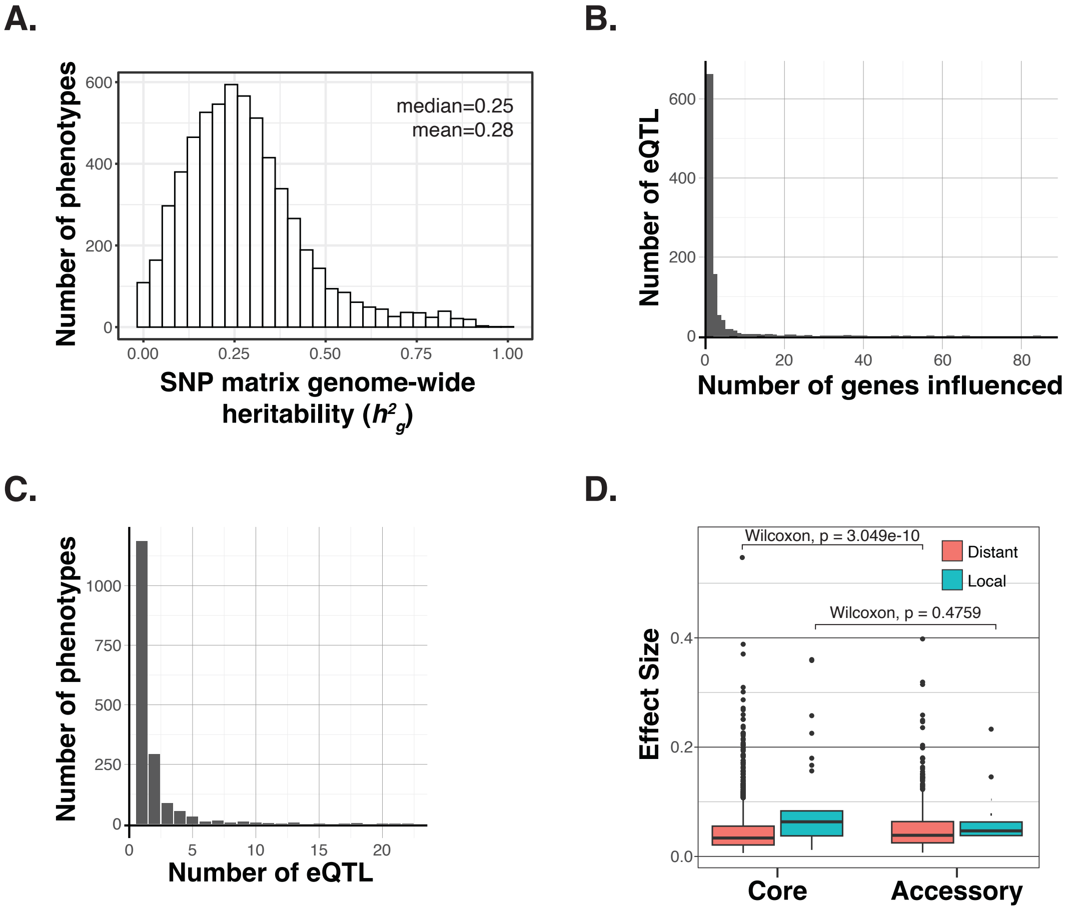

Appendix figure S2. Genome-wide heritability and GWAS results using the SNP

(A) Distribution of genome-wide heritability (h^2g) of all transcript abundance phenotypes calculated based on the kinship matrix of the hybrids. (B) Distribution of the number of genes influenced by each SNP-eQTL. (C) Distribution of the number of eQTLs associated to each gene. (D) Effect size of eQTLs based on their position, distant or local in red and cyan, and the type of gene that is influenced (core or accessory gene). Significant difference is observed only between the distant eQTLs impacting core and accessory genes (two-sided Mann-Whitney-Wilcoxon test, p-value = 3.049e-10). Quantiles for local eQTL effect sizes for core genes (n = 31): min = 0.012, 1st quartile = 0.040, median = 0.056, 3rd quartile = 0.081, max = 0.360. Quantiles for local eQTL effect sizes for accessory genes (n = 5): min = 0.034, 1st quartile = 0.035, median = 0.105, 3rd quartile = 0.192, max = 0.257. Quantiles for distant eQTL effect sizes for core genes (n = 2,555): min = 0.006, 1st quartile = 0.021, median = 0.034, 3rd quartile = 0.056, max = 0.547. Quantiles for distant eQTL effect sizes for accessory genes (n = 397): min = 0.009, 1st quartile = 0.028, median = 0.044, 3rd quartile = 0.068, max = 0.319.

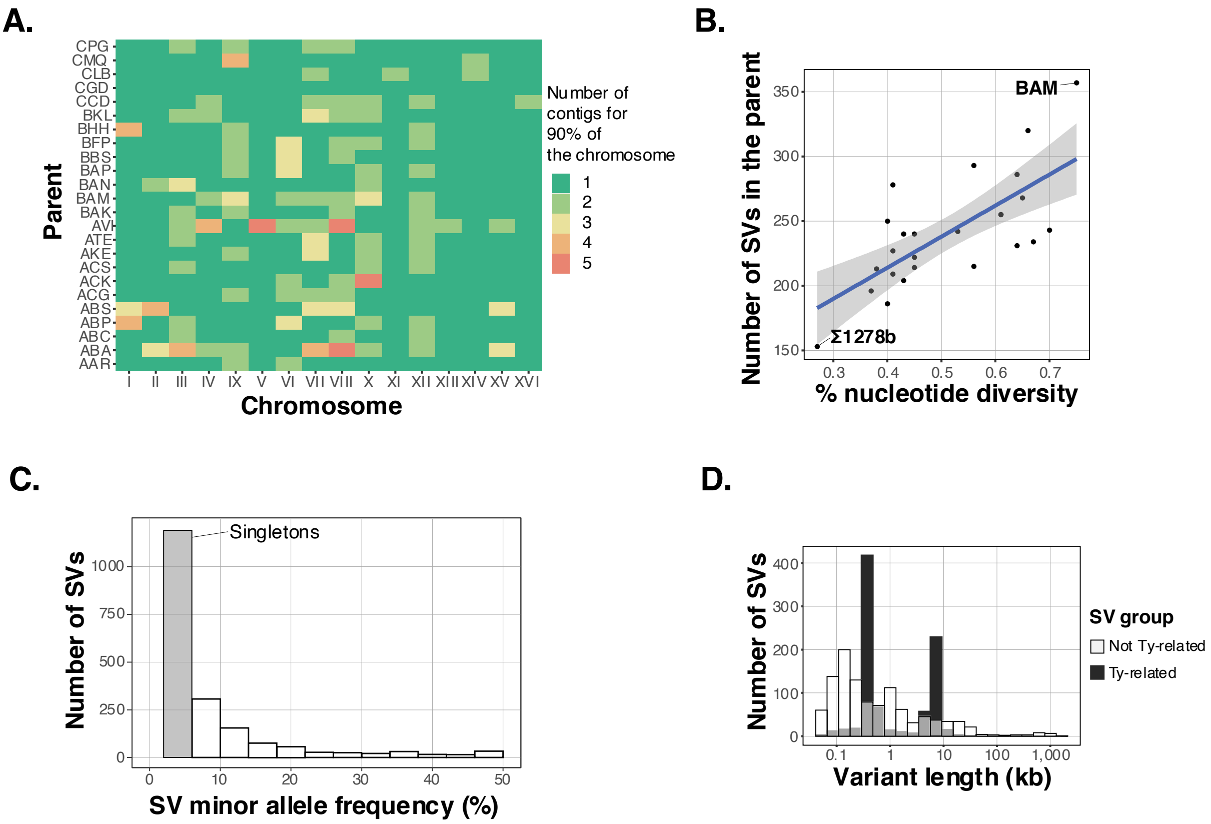

Appendix figure S3. Genome assemblies and structural variant detection statistics

(A) Number of contigs, obtained by the genome assemblies of the parents, needed to cover more than 90% of each chromosome's length. (B) Pearson's correlation between the number of SVs detected in each parent and the parents nucleotide diversity from the reference strain (S288c) (Pearson's correlation R=0.7, p-value=7.3e-5). The blue line represents the linear regression line between the two variables. (C) Distribution of the SVs' MAF across the 26 parental isolates. The grey bar highlights the SVs present in only one individuals (singletons) (D) Comparison of the variant length distributions of the Ty-related SVs (black bars) and not Ty-related SVs (white bars).

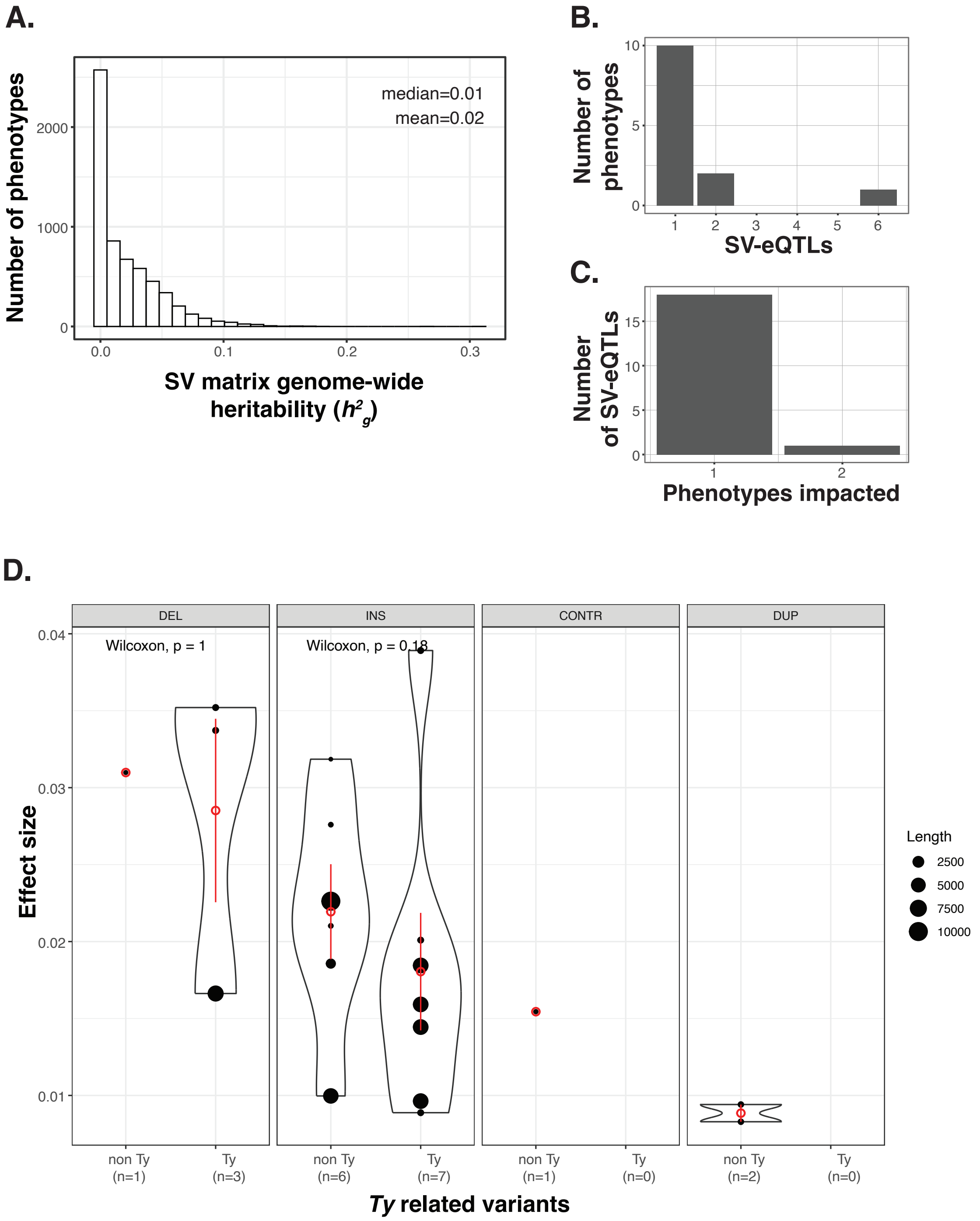

Appendix figure S4. SV genome-wide heritability and GWAS results

(A) Distribution of genome-wide heritability (h^2g) based on the SV genotype matrix, excluding the SVs present in only one of the parents. (B) Distribution of the number of eQTLs associated to each phenotype. (C) Distribution of the number of phenotypes associated to each eQTL. (D) Effect sizes for *Ty* and non-*Ty* related SVs across variant types. Error bars correspond to mean standard error from bootstrap.
